# Intermittent, low dose carbon monoxide exposure enhances survival and dopaminergic differentiation of human neural stem cells

**DOI:** 10.1371/journal.pone.0191207

**Published:** 2018-01-16

**Authors:** Nanna Dreyer-Andersen, Ana Sofia Almeida, Pia Jensen, Morad Kamand, Justyna Okarmus, Tine Rosenberg, Stig Düring Friis, Alberto Martínez Serrano, Morten Blaabjerg, Bjarne Winther Kristensen, Troels Skrydstrup, Jan Bert Gramsbergen, Helena L. A. Vieira, Morten Meyer

**Affiliations:** 1 Department of Neurobiology Research, Institute of Molecular Medicine, University of Southern Denmark, Odense, Denmark; 2 Instituto de Biologia Experimental e Tecnológica (IBET), Oeiras, Portugal; 3 Instituto de Tecnologia Química e Biológica (ITQB), Oeiras, Portugal; 4 CEDOC, NOVA Medical School/Faculdade de Ciência Médicas, Universidade Nova de Lisboa, Lisboa, Portugal; 5 Department of Pathology, Odense University Hospital, Denmark & Department of Clinical Research, University of Southern Denmark, Odense, Denmark; 6 Center for Insoluble Protein Structures (inSPIN), Department of Chemistry, Aarhus University, Aarhus, Denmark; 7 Department of Molecular Biology and Center of Molecular Biology Severo Ochoa, University Autonoma Madrid-C.S.I.C Campus Cantoblanco, Madrid, Spain; 8 Department of Neurology, Zealand University Hospital, Roskilde, Denmark; Faculty of Biochemistry, Biophysics and Biotechnology, Jagiellonian University, POLAND

## Abstract

Exploratory studies using human fetal tissue have suggested that intrastriatal transplantation of dopaminergic neurons may become a future treatment for patients with Parkinson’s disease. However, the use of human fetal tissue is compromised by ethical, regulatory and practical concerns. Human stem cells constitute an alternative source of cells for transplantation in Parkinson’s disease, but efficient protocols for controlled dopaminergic differentiation need to be developed. Short-term, low-level carbon monoxide (CO) exposure has been shown to affect signaling in several tissues, resulting in both protection and generation of reactive oxygen species. The present study investigated the effect of CO produced by a novel CO-releasing molecule on dopaminergic differentiation of human neural stem cells. Short-term exposure to 25 ppm CO at days 0 and 4 significantly increased the relative content of β-tubulin III-immunoreactive immature neurons and tyrosine hydroxylase expressing catecholaminergic neurons, as assessed 6 days after differentiation. Also the number of microtubule associated protein 2-positive mature neurons had increased significantly. Moreover, the content of apoptotic cells (Caspase3) was reduced, whereas the expression of a cell proliferation marker (Ki67) was left unchanged. Increased expression of hypoxia inducible factor-1α and production of reactive oxygen species (ROS) in cultures exposed to CO may suggest a mechanism involving mitochondrial alterations and generation of ROS. In conclusion, the present procedure using controlled, short-term CO exposure allows efficient dopaminergic differentiation of human neural stem cells at low cost and may as such be useful for derivation of cells for experimental studies and future development of donor cells for transplantation in Parkinson’s disease.

## Introduction

Parkinson’s disease is a neurodegenerative disorder affecting more than six million people worldwide [1]. The disease is associated with a progressive loss of midbrain dopaminergic neurons and subsequent depletion of striatal dopamine. Cardinal symptoms include bradykinesia, rigidity, tremor and postural instability, but non-motor symptoms also occur [2].

Several explorative clinical studies using human fetal ventral mesencephalic tissue have indicated that intrastriatal transplantation may become a future treatment for Parkinson’s disease [3–8]. However, the use of human fetal tissue is hampered by ethical concerns, suboptimal survival of grafted dopaminergic neurons, development of postgrafting dyskinesias in some patients, and the logistics related to collection and storage of the donor tissue [5,8–13].

Pre-differentiated induced pluripotent stem cells, embryonic stem cells and NSCs represent potential alternative sources of cells for cell replacement therapy in Parkinson’s disease. NSCs are self-renewable multipotent cells that can be isolated from the developing and mature nervous system. Such cells may have significant advantages compared to human fetal tissue as they can be propagated to almost unlimited numbers of relatively homogenous cells *in vitro* and frozen without significant loss of viability [14,15]. Nevertheless, efficient, simple and cost-effective protocols for controlled generation of functional dopaminergic neurons are still not available.

CO is an endogenous product of heme degradation, a reaction catalyzed by the enzyme heme oxygenase [16]. This gasotransmitter shows several beneficial biological activities and has been the target of extensive studies in relation to cardiovascular diseases, inflammatory disorders and organ transplantation [17]. The great potential of CO in biomedical applications has prompted development of several delivery strategies of CO for therapeutic or research purposes. Gas inhalation is the most simple strategy and has been greatly used in pre-clinical *in vivo* experiments [18–20]. Cell cultures can also be exposed to CO in gas chambers as described for neurons [21] and macrophages [22]. Another possible strategy for *in vitro* application of CO is the use of CO-saturated solutions [23,24]. Nevertheless, for all these approaches CO gas bottles are handled with the potential risk of leaking the odorless and highly toxic gas. Furthermore, gas inhalation is not the most appropriate method for CO administration in a clinical context, since it promotes increased carboxyhaemoglobin levels as well as CO delivery to both healthy and diseased tissues. Therefore, CO-releasing molecules (CORMs) providing controlled CO delivery have been developed [25]. The most studied non-metal based CORM is boranocarbonate [H_3_BCO_2_]Na_2_ (CORM-A1), which in several studies has been shown to modulate cytoprotection, hormesis and inflammation [26–28]. There are also many metal-based compounds studied in biological systems, and the most explored is the water-insoluble dimer [Ru(CO)_3_Cl_2_]_2_ (CORM-2) and its water soluble derivative Ru(CO)_3_Cl(κ^2^-H_2_NCH_2_CO_2_) (CORM-3). CORM-2 and CORM-3 have been tested in pre-clinical studies of cardioprotection [29,30], inflammation [31–33], neuroprotection [34–36], transplantation [37] and pain [38].

In the CNS, the CO/heme oxygeanse axis is a key player in processes involved in cytoprotection, vasomodulation, neuroinflammation, cell death, metabolism and cellular redox responses [39]. CO was first recognized as a neurotransmitter by Verma and colleagues [40], and their work led to extensive research on CO and heme oxygenase in the nervous system. Interestingly, both heme oxygenase and exogenous administration of CO were reported to stimulate neuroprotection and maintenance of tissue homeostasis in response to various pathophysiological conditions; including cerebral ischemia [20,36,41–43], cerebrovasodilation [28,44,45], neuroinflammatory [19,34,35,46], and neurodegenerative diseases [47–49].

The CO-induced pathways and putative targets are a matter of debate. Nevertheless, it is well accepted that CO activates soluble guanylyl cyclase and nitric oxide synthase, increasing the cGMP and nitric oxide levels, respectively, whose best described effects are modulation of vasodilation [50]. In neurons, CO-induced cGMP production is involved in protection against cell death [21,36,51]. Nitric oxide signaling is related to anti-inflammatory effect of CO in microglia [32].

In CO pathways, low amounts of reactive oxygen species play a crucial role in preconditioning and cytoprotection in neurons and astrocytes [21,24]. Interestingly, Chin and colleagues have demonstrated CO-mediated stabilization of HIF-1α [52], although it is a controversial subject [53].

In the present study two major novelties are approached. Firstly, the potential effect of CO on dopaminergic differentiation of human NSCs is assessed. Secondly, a new strategy for delivering CO gas is being tested. In this new system, CO is generated by a decarbonylation reaction using the new CORM methyldiphenylsilacarboxylic acid (MePh_2_SiCO_2_H), along with the non-transition-metal activator potassium fluoride and dimethyl sulfoxide [54]. This strategy avoids the use of CO gas bottles, thus being safer and more cost-effective than previously described methods.

## Materials and methods

### Carbon monoxide releasing molecules (CORMs)

**Fig 1:**
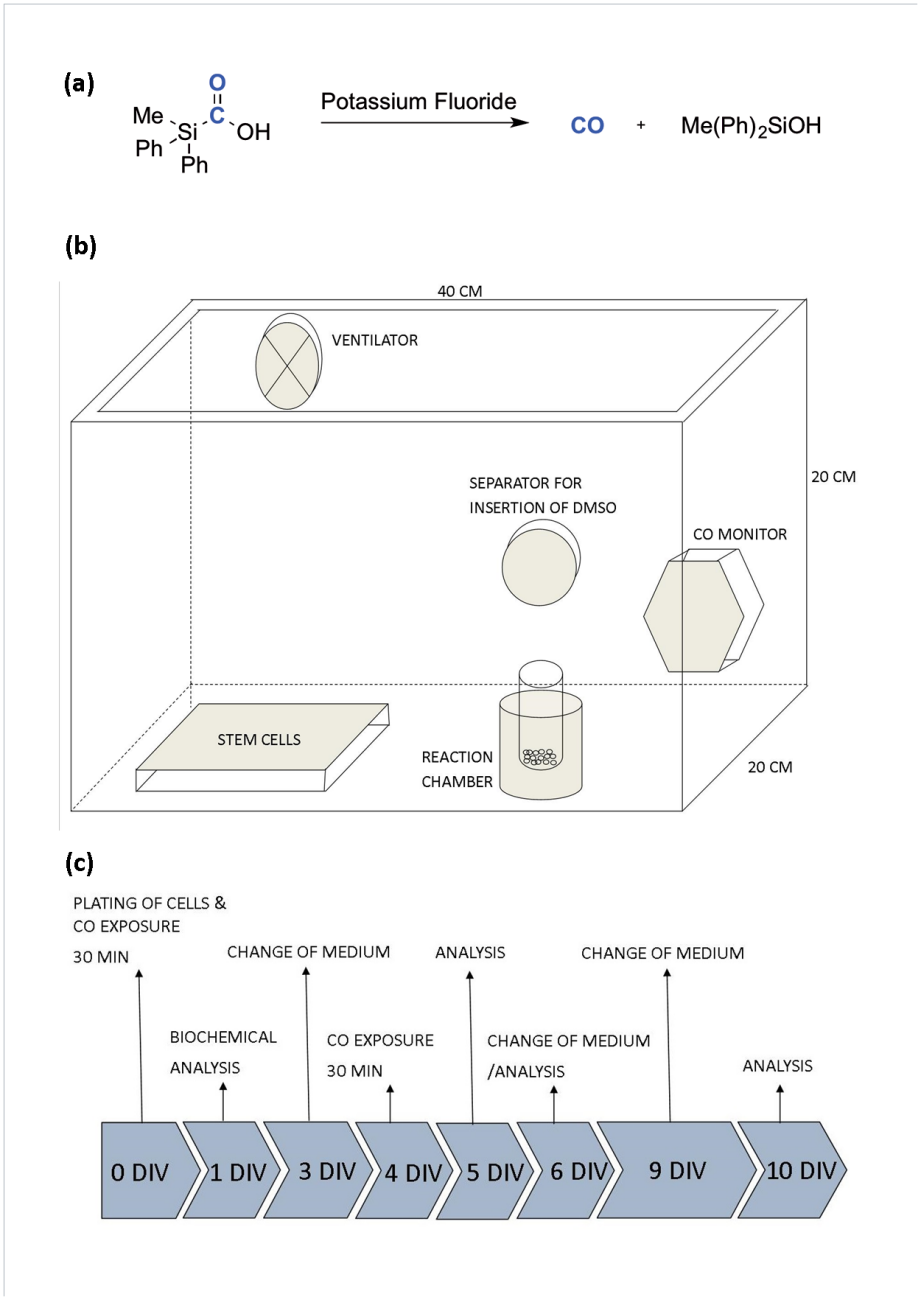
Chemical reaction releasing carbon monoxide (CO) and experimental setup. (a) The chemical reaction releasing CO when mixing methyldiphenylsilacarboxylic acid (MePh_2_SiCO_2_H), potassium fluoride and dimethyl sulfoxide. (b) Illustration of the CO gas chamber. (c) Human neural stem cells were plated at day 0, cultured for 4 hrs followed by one or two 30 min CO treatments. All culture medium was changed at days 4, 6 and 9. hREN VM cell cultures received CO treatment at days 0 and 4 and were used for immunocytochemistry and Western blotting after 6 days. For experiments with hVMbclX_L_ cells: 1) cultures received CO treatment at day 0 followed by immunocytochemistry at days 1, 6 and 10, or 2) cultures received CO treatment at days 0 and 4 and were used for cytokine profiling (day 5), immunocytochemistry (day 6 and 10), Western blotting (day 6) or MTS assay (day 6). Untreated control cultures were included in all experiments. DIV = days *in vitro*.

CORMs are chemical compounds typically containing transition-metal carbonyl complexes that can release CO under certain conditions [55]. We used a crystalline silacarboxylic acid, which was synthesized from the corresponding chlorosilane via reduction with metallic lithium, and allowed it to react with CO_2_ [54]. By mixing methyldiphenylsilacarboxylic acid (MePh_2_SiCO_2_H) with the non-transition-metal activator potassium fluoride (Sigma) and the solvent dimethyl sulfoxide (Sigma) a decarbonylation reaction results in CO-release (Fig 1a) [54]. For the present study, a plexi-glass chamber was developed (Fig 1b). In order to achieve controlled CO concentrations we used 1 mg MePh_2_SiCO_2_H, 0.3 mg potassium fluoride and 62.5 μl dimethyl sulfoxide per mg MePh_2_SiCO_2_H to generate 7.4 ppm CO in the chamber. The amount of solids required to achieve a predefined level of CO (12,5–100 ppm) were placed in a glass vial (Supelco) and transferred to the exposure chamber together with the culture plates/flasks (none of the solids entered the culture medium). The CO concentration in the chamber was monitored with a Dräger Pac 7000 CO sensor device (Dräger Safety AG & Co. KGaA, Lübeck, Germany). The chamber was placed at 36°C, 5% CO_2_ and 95% humidified air. To start CO release, dimethyl sulfoxide was lead through a separator in the wall of the chamber and into the vial with silacarboxylic acid/potassium flouride. A ventilator homogenized the concentration of gas in the closed atmosphere (Fig 1b). The CO concentration was measured throughout all experiments (S1 Fig).

### Culturing and passaging of NSCs

Tissue procurement was in accordance with the Declaration of Helsinki and in agreement with national and institutional rules as well as the ethical guidelines of Network of European CNS Transplantation and Restoration (NECTAR).

Two human ventral mesencephalic (VM) stem cell lines generated in previous studies were used (hVMbclX_L_; hReN). In brief, VM cells were derived from a 10-week-old foetus and immortalized using a retroviral vector coding for *v-myc* (LTR-vmyc-SV40p-Neo-LTR), creating a multipotent cell line (hVM1) [56]. The hVM1 cells were genetically modified (MLV-based retroviral vector) to over-express the anti-apoptotic gene BclX_L_ (LTR-Bcl-X_L_-IRES-rhGFP-LTR), essentially as described by Liste *et al*. [57].

Cells were propagated in poly L-lysine (10 μg/ml; Sigma)-coated culture flasks containing HNSC100 medium (Dulbecco’s modified Eagle’s medium F12 w. Glutamax (Gibco), 2% (v/v) 30% glucose (Sigma), 0.5% (v/v) 1 M Hepes (Gibco), 2.5% (v/v) AlbuMAX-I (Gibco), 1% (v/v) N2 supplement (Gibco), 1% (v/v) NEAA (Sigma) and 1% penicillin/streptomycin (Gibco)) supplemented with 20 ng/ml epidermal growth factor (R&D Systems) and 20 ng/ml basic fibroblast growth factor (R&D Systems) at 36°C, 5% CO_2_/95% humidified air. Medium was changed every third day, and cells passaged at 80% confluence. Cells were counted using an automatic cell counter (S2 Fig).

The hREN VM cell line was derived from a 10-week-old foetus (ReNeuron; Millipore) and immortalized by retroviral transfection with the oncogene *v-myc* [58]. hREN VM cells were cultured as described above.

### Neuronal differentiation protocols

NSCs were passaged and plated into poly L-lysine-coated 24-/96-well trays or T75 culture flasks (Nunc, Sigma) with HNSC100 medium (26,000 cells/cm^2^). Both cell lines, hVMbclX_L_ (passage 26–29) and hREN VM (passage 7), were exposed to CO for 30 min (hVMbclX_L_ cells also for 45 and 60 min). Untreated cultures served as controls.

hVMbclX_L_ cultures either received CO treatment at day 0 followed by differentiation for 1, 6 and 10 days or were exposed to CO at days 0 and 4 and differentiated until day 6 or 10 (Fig 1c).

hREN VM cells received CO treatment at days 0 and 4 and were differentiated until day 6. The culture medium was changed every third day.

### Neurospheres

Cells (hVMbclX_L_) were plated (233,000 cells/ml medium) in 35 mm petri dishes (Nunc; Sigma) with 4.3 ml HNSC100 medium containing 20 ng/ml epidermal growth factor and basic fibroblast growth factor (R&D Systems) and grown at 36°C in 5% CO_2_/and 95% humidified air. Resulting neurospheres received 25 ppm CO for 30 min at days 0 and 4 versus untreated controls. At day 9, all neurospheres were processed for immunohistochemistry.

### Fixation and immunocytochemistry

Monolayer cultures were fixed (20 min) in 4% paraformaldehyde/0.15M phosphate buffer. For immunocytochemistry cultures were washed in 0.05M tris-buffered saline (TBS) containing 0.1% triton X-100 (Sigma) and pre-incubated (30 min) in TBS/10% donkey or sheep serum (Gibco). Primary antibodies (24 hrs; 4°C) were diluted in TBS/10% donkey or sheep serum: Tyrosine hydroxylase (TH; polyclonal rabbit; Chemicon) 1:600; β-tubulin III (β-tubIII; monoclonal mouse; Sigma) 1:2000; human nuclei (HN; monoclonal mouse; Chemicon) 1:500; microtubule associated protein 2ab (MAP2; monoclonal mouse; Sigma) 1:2000; Ki67 (monoclonal mouse; BD Pharmigen) 1:500; active/cleaved caspase3 (Casp3; polyclonal rabbit; R&D Systems) 1:5000.

Cultures were then incubated for 1 hr with biotinylated anti-rabbit or anti-mouse antibodies (GE Healtcare) diluted 1:200 in TBS/10% donkey or sheep serum followed by 1 hr with horseradish peroxidase-conjugated streptavidin (GE Healthcare) diluted 1:200 in TBS/10% donkey or sheep serum. For development/visualization 3,3´-diaminobenzidine (Sigma) was used.

Neurosphere cultures were fixed (24 hrs) in 4% neutral buffered formalin (Bie&Berntsen), washed in a NaCl followed by treatment with plasma and thrombin (3:2 ratio). The resulting fibrin-clot was paraffin embedded and sectioned at 3 μm. Sections were dewaxed in Xylene (DAKO) and rehydrated in a graded series of ethanol. Endogenous peroxidase was inhibited by 1.5% hydrogen peroxide/TBS (DAKO). Heat-induced epitope retrieval (DAKO) was performed with tris-EDTA-glucose (DAKO) or target retrieval solution (DAKO) buffer (microwave: 9 min at 900 W, 15 min at 440 W—subsequently 15 min at room temperature). Afterwards sections were placed in an Autostainer Universal Staining System (DAKO) for 1 hr. HIF1α (1:1000; B&D Systems) in target retrieval solution buffer and carbonic anhydrase IX (CA9; 1:1000; Novus Biologicals) in cell conditioning1 buffer. Sections were incubated for 30 min with secondary antibodies; Powervision and Optiview for HIF1α and CA9, respectively. Visualization with 3,3´-diaminobenzidine was followed by staining with Mayer’s Hematoxylin (DAKO).

### Western blotting

Western blotting was performed as described by Krabbe *et al*. [59]. Membranes were incubated (over night/4°C) with anti-TH (1:2000; monoclonal mouse; Chemicon) or anti-β-tubIII antibody (1:2000; monoclonal mouse; Sigma) diluted in TBS/Tween-20, washed, incubated (1 hr) with horseradish peroxidase-conjugated anti-mouse antibody (1:2000; DAKO) diluted in TBS/Tween-20, developed with chemiluminiscence (SuperSignal^®^Extended duration substrate; Thermo Scientific), and visualized using a charge coupled device camera. Loading control: alpha-actin antibody (1:6000; mouse; Chemicon).

### Quantitative-Polymerase chain reaction

Messenger RNA was extracted using the High Pure RNA isolation kit (Roche Diagnostics), and cDNA synthesis was performed using the Transcriptor High Fidelity cDNA synthesis kit (Roche Diagnostics). PCR was performed using specific forward and reverse primers designed for: TH (5′-CGGGCTTCTCGGACCAGGTGTA-3′ and 5′-CTCCTCGGCGGTGTACTCCACA-3′), Nurr1 (5′-CTGCAAAAGGAGACAATATAGACCA-3′ and 5′-ATCGTAGACCCCAGTCACATAA-3′), Dopamine transporter (DAT; 5′-TTCCTCAACTCCCAGTGTGC-3′ and 5′-AGGATGAGCTCCACCTCCTT-3′), Dopamine beta-hydroxylase (DBH; 5′-CTTCCTGGTCATCCTGGTGG-3′ and 5′-TCCAGGGGGATGTGATAGGG-3′) and ribosomal protein L22 (5’-CACGAAGGAGGAGTGACTGG-3’ and 5’-TGTGGCACACCACTGACATT-3’). Fast Start DNA Master Plus SYBR Green I (Roche Diagnostics) was applied using the following protocol: denaturation program, 95°C for 10 min followed by 45 cycles of 95°C for 10 sec, 60°C for 10 sec and 72°C for 10 sec.

### MTS cell viability assay

Metabolically active, viable cells undergoing proliferation were investigated using the MTS kit (CellTiter 96^®^Aq_ueous_One Solution; Promega) according to the manufacturer’s instructions and a Vmax kinetic microplate reader with SoftMax^®^Pro software (Molecular Devices).

### High-performance liquid chromatography

Dopamine and homovanillic acid were assessed in culture medium/extracts derived from cells differentiated (14 days) according to our standard protocol supplemented by 25 ppm CO (30 min) at days 0 and 4.

*Sample preparation; medium*: Cells were washed twice in Hank’s balanced salt solution (Life Technologies), followed by incubation (2 hrs/36°C) in 200 μl of Hank’s balanced salt solution containing 10μM nomifensine (Research Biochemicals International). A 100 μl sample was transferred to HPLC vials containing 50 μl of mobile phase (10% methanol (v/v), 20 g/l citric acid monohydrate, 100 mg/l octane-1-sulfonic acid sodium salt, 40 mg/l EDTA dissolved in Milli-Q water and pH adjusted to 4.0; all from Merck/VWR Chemicals) and stored at -20°C until HPLC analysis with electrochemical detection [60,61].

*Sample preparation; extracts*: After removing the culture medium, 150 μl/well of 0.1 M perchloric acid (Merck) with antioxidants (0.2 g/L Na_2_S_2_O_5_, 0.05g/L Na_2_-EDTA; Merck) was added. Cells were resuspended in perchloric acid, transferred to dark eppendorf vials on ice, briefly sonicated and centrifugated (20.000 x *g*/20 min/4°C). The supernatant was stored at -20°C until analysis.

### Multi cytokine array

Conditioned culture medium was frozen (-20°C), and cells were collected as described for Western blotting but with the cell pellets dissolved in RayBio^®^ Cell Lysis Buffer (RayBioech). Protein concentrations were determined using a protein assay (BioRad). Four membranes (Human Cytokine Antibody Array-5; RayBiotech) were incubated (30 min/room temperature) with blocking buffer (RayBiotech), and 1 ml conditioned culture medium or 160 μg cell lysate (diluted to 1 ml in blocking buffer) was added (incubation; 1 hr/RT followed by 12 hrs/4°C). After washing, membranes were incubated with biotin-conjugated antibody diluted in blocking buffer (2 hrs/room temperature and 12 hrs/4°C). Membranes were then incubated with horseradish peroxidase-conjugated streptavidin diluted in blocking buffer (2 hrs/room temperature), washed, developed with chemiluminiscence (RayBiotech), and visualized using a charged coupled device camera (Carestream). Densitometric analysis was performed using Image J software (NIH). Changes >50% relative to control were taken into consideration.

### Measurement of reactive oxygen species (ROS)

Determination of ROS in cultured cells was performed by analysis of hydrogen peroxide (H_2_O_2_) formation. H_2_O_2_ production was measured with a homogenous bioluminescence ROS-Glo^™^H_2_O_2_ Assay Kit according to the manufacturer’s protocol (Promega). Briefly, cells were seeded in 96-well plates (5.000 cells/ well). ROS levels were determined at day 0 (two hrs after after the first CO exposure) and at day 6 *in vitro* (two days after the second CO exposure). The ROS-Glo^™^H_2_O_2_ Substrate was added during treatment (final concentration 25 μM), and the cells were incubated for an additional hours (37°C, CO_2_ incubator). After incubation, 50 μl medium from each well was transferred to 96-well plates. ROS-Glo^™^H_2_O_2_ Detection Solution was added (incubation for 20 min) before luminescence was determined using an Orion L Microplate Luminometer (Titertek Berthold). Luminescence signals were normalized to protein concentrations determined by the BCA Protein Assay Kit (Thermo Fisher Scientific).

### Cell counting

Quantification of cells was performed using bright field microscopy (Olympus). Cells with an extensive immunostaining and a well-preserved cellular structure were counted in 16 randomly selected areas/well (X200) using an ocular grid (0.5x0.5 mm^2^).

### Statistical analysis

Statistical analysis was performed using Prism GraphPad Software. Sample size estimates were made by power analysis. Cell numbers were compared by one-way analysis of variance (ANOVA) followed by Dunnett’s multiple comparisons test. Student’s t-test or the non-parametric Mann-Whitney U-test was used (depending on data distribution) when comparing only two groups. *p*<0.05 (*), *p*<0.01 (**) and *p*<0.001 (***).

## Results

### Carbon monoxide release

To characterize and validate the reaction from the new CO-releasing molecule (CORM) MePh_2_SiCO_2_H (Fig 1a), the CO concentration was measured in the gas chamber every minute throughout a 30 min exposure period (Fig 1b). The CO level increased rapidly after mixing MePh_2_SiCO_2_H, potassium fluoride and dimethyl sulfoxide, reaching the desired concentrations after 5 min and maintaining a constant level during the entire exposure period (S1 Fig). Calculations of CO concentrations, using available data on the CO (g) solubility in water at 36°C and 1 atmosphere, revealed relatively low levels of CO in the culture medium, e.g. 25 ppm CO in the gas chamber would result in approximately 20 nM CO in the medium.

### Effect of CO on stem cell differentiation

**Fig 2:**
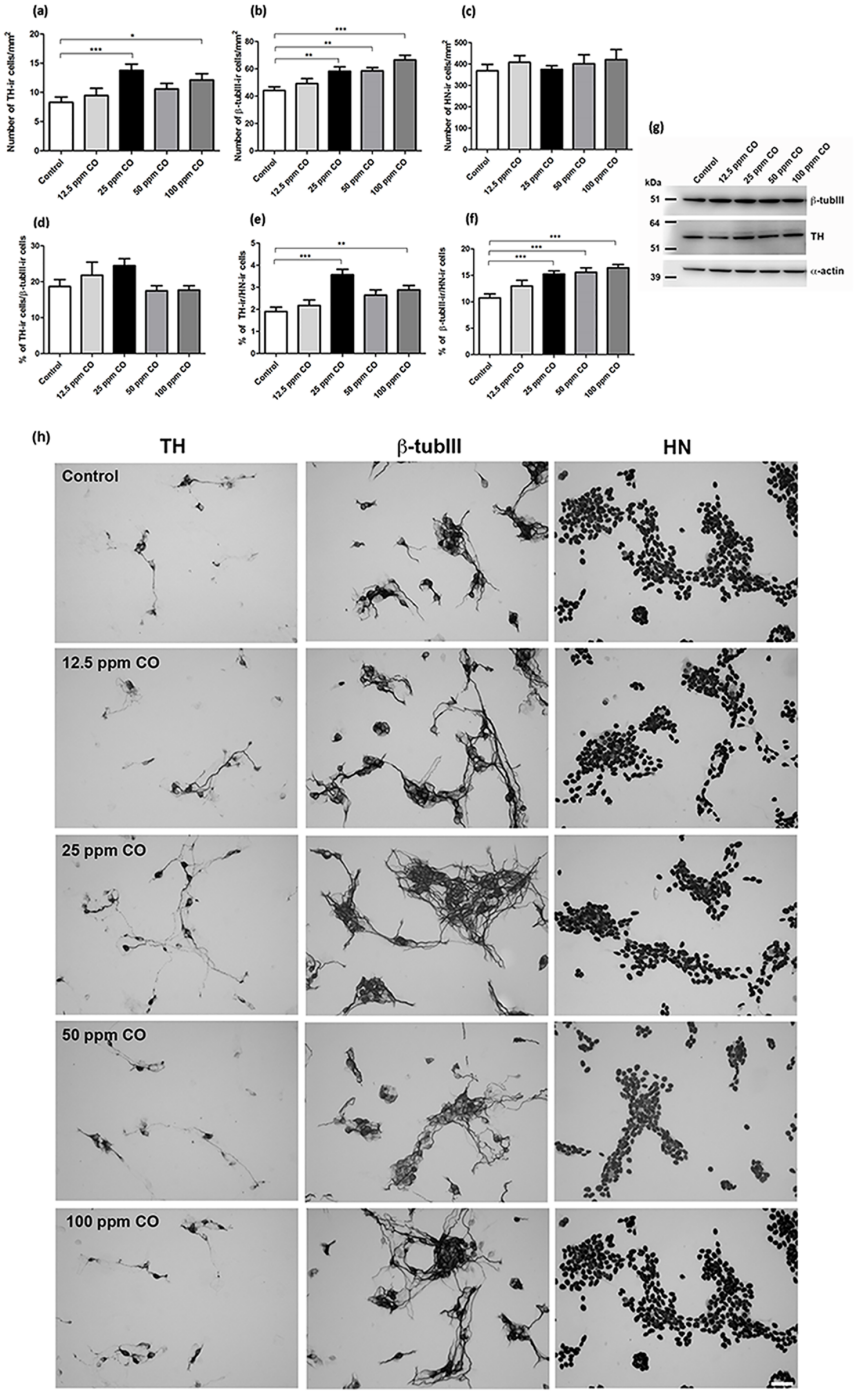
Dose response effects of short-term carbon monoxide (CO) treatment on neuronal differentiation of human neural stem cells (hVMbclX_L_). Quantitative analysis of total cells (human nuclei-immunoreactive (HN-ir) cells), cells differentiated into tyrosine hydroxylase-ir (TH-ir) and β-tubulinIII-ir (β-tubIII) neurons in 6-day-old cultures. Cultures received CO treatment (12.5–100 ppm) for 30 min at days 0 and 4. Untreated cells served as controls. (a) Quantification of TH-ir neurons showed a significant increase for 25 and 100 ppm CO compared to control. (b) Quantification of β-tubIII-ir neurons revealed a significant increase for cells treated with 25, 50 and 100 ppm CO. (c) No differences between numbers of HN-ir cells were seen. (d) The percentage of TH-ir neurons of β-tubIII-ir neurons did not differ between the groups. (e) Exposure to 25 and 100 ppm CO resulted in a significant increase in the percentage of TH-ir neurons of total cells, and (f) 25–100 ppm CO resulted in a significant increase in the percentage of β-tubIII-ir neurons of total cells as compared to untreated controls. Data are based on four independent experiments (n = 29-40/group) and expressed a mean±SEM (*p<0.05, **p<0.01, ***p<0.001). (g) Western blotting for β-tubIII showed an increase in signal intensities for all CO treatment groups compared to control. 10 μg protein was loaded per lane and α-actin served as loading control. β-tubIII ≈ 50 kDa; TH ≈ 56 kDa; α-actin ≈ 43 kDa. (h) Digital images of cultures treated with CO expressing TH, β-tubIII and HN. Scale bar = 50 μm.

To investigate the effect of CO on the dopaminergic differentiation, hVMbclX_L_ cells were differentiated for 6 days and received CO (12.5–100 ppm; 30 min) at days 0 and 4 (Fig 1c). The density of TH-ir neurons increased significantly, when the cells were exposed to CO at 25 and 100 ppm compared to control (control = 8.3±0.9; 12.5 ppm CO = 9.5±1.2; 25 ppm CO = 13.8±1.1 (p<0.001); 50 ppm CO = 10.6±1.0; 100 ppm CO = 12.1±1.1 (p<0.05); TH-ir cells/mm^2^; mean±SEM; n = 29–40; four independent experiments) (Fig 2a). Moreover, the percentage of TH-ir neurons relative to HN-ir cells (total cells) was significantly higher for cultures exposed to CO at 25 and 100 ppm compared to control (control = 1.9±0.2; 12.5 ppm CO = 2.1±0.2; 25 ppm CO = 3.6±0.2 (p<0.001); 50 ppm CO = 2.6±0.2; 100 ppm CO = 2.8±0.2 (p<0.05); % TH-ir cells of total cells; mean±SEM; n = 29–40; four independent experiments) (Fig 2e). Representative digital images visualizing the content and morphology of TH-ir neurons are shown in Fig 2h.

The density of β-tubIII-ir neurons increased significantly in cultures treated with 25–100 ppm CO compared to control (control = 44.2±2.8; 25 ppm CO = 58.3±3.1 (p<0.01); 50 ppm CO = 58.5±2.3 (p<0.01); 100 ppm CO = 66.5±3.4 (p<0.001); β-tubIII-ir cells/mm^2^; mean±SEM; n = 29–40; four independent experiments) (Fig 2b). Furthermore, the percentage of β-tubIII-expressing neurons of total cells was significantly higher (control = 10.7±0.8; 25 ppm CO = 15.2±0.6 (p<0.001); 50 ppm CO = 15.6±0.8 (p<0.001); 100 ppm CO = 16.4±0.6 (p<0.01); % β-tubIII-ir cells of total cells; mean±SEM; n = 29–40; four independent experiments) (Fig 2f). No differences in total cells were detected (Fig 2c). Representative images of β-tubIII-ir and HN-ir cells are shown in Fig 2h.

TH and β-tubIII expression was also investigated by Western blotting showing increased TH expression at 25 and 100 ppm CO compared to control. Moreover, there was indication of increased β-tubIII expression in all CO treated groups (Fig 2g).

To investigate whether similar effects could be obtained for other cell lines, hREN VM cells were treated with 25 ppm CO at days 0 and 4. A significantly higher density of β-tubIII-ir neurons was detected for CO treated cultures compared to control (control = 2.9±0.3; CO = 5.7±0.4 (p<0.001); β-tubIII-ir cells/mm^2^; mean±SEM; n = 10) (S3a Fig). Total cell numbers did not differ between the groups (control = 418±9.7; CO = 439±20; HN-ir cells/mm^2^; mean±SEM; n = 4). Moreover, the percentage of β-tub III-ir neurons relative to total cells was significantly higher for the CO group compared to control (control = 0.7±0.07; CO = 1.3±0.08 (p<0.001); % β-tubIII-ir cells of total cells; n = 10) (S3b Fig). Representative digital images of β-tubIII-ir neurons and HN-ir cells are shown in S3c and S3d Fig. Western blotting analysis for β-tubIII did not reveal differences in signal intensities between CO treated cultures and controls. The number of TH-ir neurons was too low in the hREN VM cell cultures for a valid comparison of the groups.

In summary, short-term CO exposure during stem cell differentiation has the capacity to increase both density and relative content of TH-ir and β-tubIII-ir cells.

### Effect of short versus longer-term CO exposure and role of differentiation time

To investigate the potential role of short-term versus longer-term CO exposure, hVMbclX_L_ cells were treated with 25 ppm CO for 30, 45 or 60 min at day 0 and 4 and differentiated for 6 days (n-16/group; two independent experiments). All treatments resulted in significantly higher relative contents of TH-ir cells, but no differences between the CO exposure groups were detected (data not shown).

**Fig 3:**
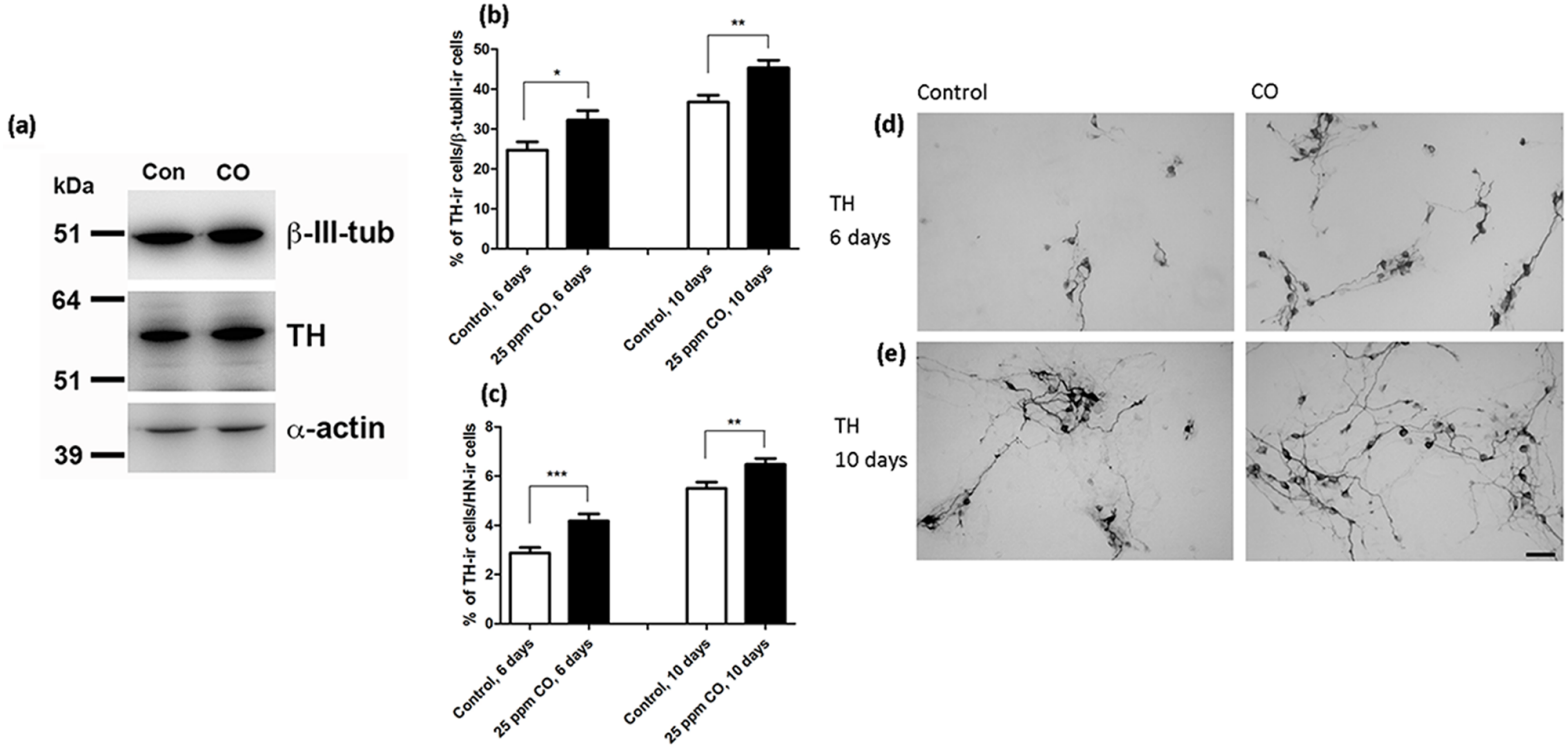
Effects of carbon monoxide (CO) on neuronal and dopaminergic differentiation. Quantitative analysis of 6- and 10-day-old cultures (hVMbclX_L_) differentiated into dopaminergic neurons by exposure to 25 parts per million (ppm) CO (30 min) at days 0 and 4. Control cells followed the same protocol but received no CO treatment. (a) Western blotting for β-tubulinIII (β-tubIII) and tyrosine hydroxylase (TH) showed a slight increase in band intensities after CO treatment compared to controls. 15 μg protein was loaded per lane and α-actin served as a loading control. β-tubIII ≈ 50 kDa; TH ≈ 56 kDa; α-actin ≈ 43 kDa. (b) At day 10, the percentages of TH-immunoreactive (-ir) neurons of total neurons (β-tubIII) were significantly higher for the CO treatment groups compared to control. (c) At days 6 and 10 also the percentages of TH-ir neurons of total cells (human nuclei (HN)-ir cells) were significantly increased for the CO treatment groups (n = 17–20; two independent experiments). Data are expressed as mean±SEM (*p<0.05, **p<0.01, ***p<0.001). (d, e) Representative digital images of TH-ir neurons displaying a mature neuronal morphology with long processes. Scale bar = 50 μm. Con = control.

To address if the effect of CO was transient or long-lasting hVMbclX_L_ cells received CO treatment (25 ppm; 30 min) at days 0 and 4 and were differentiated for 6 or 10 days (Fig 3). At day 6 and 10, the content of TH-ir neurons relative to β-tubIII-ir neurons had increased significantly in the CO treated groups (control 6 days = 24.7±2.1; 25 ppm CO 6 days = 32.2±2.4 (p<0.05); control 10 days = 36.7±1.7; 25 ppm CO 10 days = 45.3±1.9 (p<0.01); % TH-ir cells of β-tubIII-ir neurons; mean±SEM; n = 17–20; two independent experiments) (Fig 3b). Furthermore, the relative yields of TH-ir neurons of total cells had increased (control 6 days = 2.9±0.2; 25 ppm CO 6 days = 4.2±0.3 (p<0.01); control 10 days = 5.5±0.3; 25 ppm CO 10 days = 6.5±0.2 (p<0.001); % TH-ir cells of total HN-ir cells; mean±SEM; n = 17–20; two independent experiments) (Fig 3c). At day 6 and 10, Western blotting analysis indicated increased signal intensities for β-tub III, and at day 10 TH expression was slightly increased for cultures exposed to CO compared to control (Fig 3a). Representative photomicrographs of TH-ir and β-tubIII-ir neurons are shown in Fig 3d and 3e.

To investigate if a single dose of CO would be sufficient to elevate the content of TH-ir cells, a group of cultures were exposed to 25 ppm CO at day 0 followed by differentiation for 1, 6 and 10 days. No difference was found between CO treatment and control cultures at day 1, whereas a significant increase in TH-ir neurons was seen at 6 and 10 days after CO treatment (data not shown). The number of HN-ir cells did not differ between CO treatment and control cultures at any time point. Consequently, the relative content of TH-ir neurons had increased significantly at day 6 and 10 in CO exposed cultures compared to controls.

In summary, the positive effect of CO on the relative content of TH-ir cells was not transient, and it could be obtained even with a single dose of CO.

### Effect of CO on neuronal maturation and dopaminergic capacity

**Fig 4:**
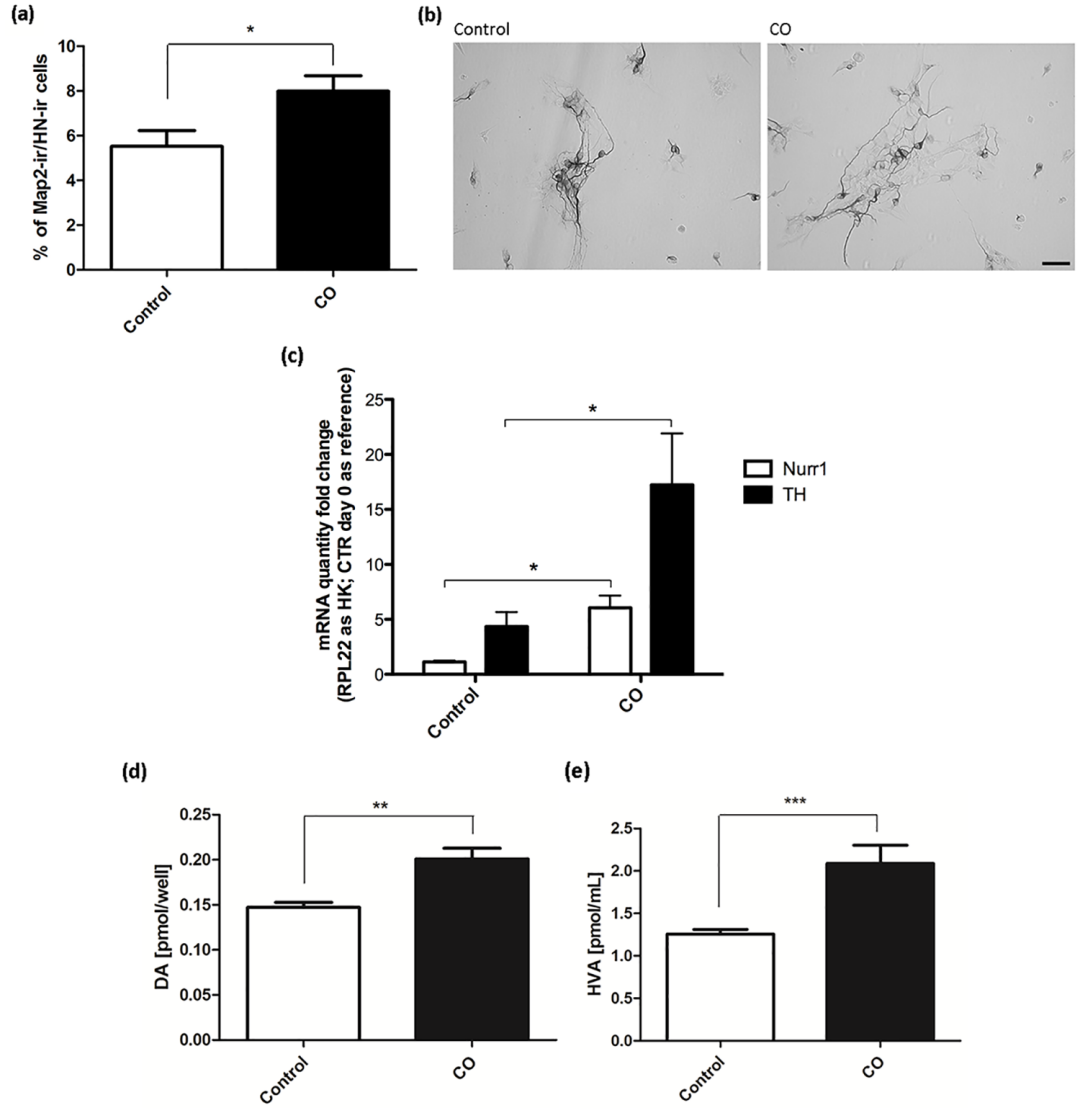
Characterization of neuronal cells in differentiated cultures. Assessment of neuronal maturation of hVMbclX_L_ cells receiving 25 parts per million (ppm) CO treatment (30 min) at days 0 and 4 and differentiated for 6, 10 and 14 days. Control cultures followed the same protocol but received no CO treatment. (a) Percentage of microtubule-associated protein2ab-immunoreactive (MAP2-ir) neurons of total cells showed a significant increase for cultures treated with CO compared to controls (control = 5.52±0.71; CO = 7.98±0.69 (p<0.05); % Map2-ir cells of total cells; mean±SEM; n = 26; two independent experiments). Data are expressed as mean±SEM. (b) Digital images of MAP2-ir neurons showing mature neuronal morphology with long processes. Scale bar = 50 μm. (c) Quantitative mRNA analysis of hVMbclX_L_ cells receiving 25 ppm CO treatment at days 0 and 4 and differentiated for 10 days. Control cells followed the same protocol but received no CO treatment. Quantities of mRNA were compared with mRNA levels at day 0. Tyrosine hydroxylase (TH) and Nurr1 mRNA levels were significantly increased for cultures treated with CO compared to controls. (d,e) HPLC analysis for dopamine (DA) in cell extracts (n = 10–11; **p<0.01) and the DA metabolite homovanillic acid (HVA) in conditioned culture medium (n = 11–12; ***p<0.001) from untreated controls and cultures receiving 25 ppm CO at days 0 and 4 and differentiated for 14 days. The analyses revealed significant elevations in both DA and HVA for cultures treated with CO compared to controls.

To investigate the potential effect of CO on neuronal maturation, numbers of mature MAP2-ir neurons were quantified in 6-day-old cultures (25 ppm CO; 30 min; day 0 and 4 versus control). The percentage of MAP2-ir neurons of total cells was significantly higher for CO-treated cultures (control = 5.5±0.7; CO = 7.9±0.7 (p<0.05); % MAP2-ir cells of total cells; mean±SEM; n = 26; two independent experiments) (Fig 4a). Moreover, CO treated cells displayed a more mature morphology with long and branching processes. Representative images of MAP2-ir neurons can be seen in Fig 4b.

The expression of catecholaminergic/midbrain-specific genes (TH, Nurr1, DAT and DBH) was assessed by mRNA quantification using real-time Q-PCR. TH and Nurr1 (Fig 4c) were increased significantly after CO treatment, whereas DAT levels were lower (not shown). DBH mRNA levels were also increased after CO treatment (not shown). HPLC analysis revealed a significant elevation of dopamine levels in cell extracts (Fig 4d) and significantly increased homovanillic acid levels in conditioned culture medium from cells exposed to CO compared to controls (Fig 4e). Noradrenaline could not be detected under the chromatographic conditions used.

In summary, CO treatment stimulates neuronal maturation and formation of neurons with midbrain characteristics.

### Mechanisms of action: Effects of CO on cell proliferation, apoptosis, cytokine profile, expression of hypoxia-inducible factor-1α, and production of ROS

**Fig 5:**
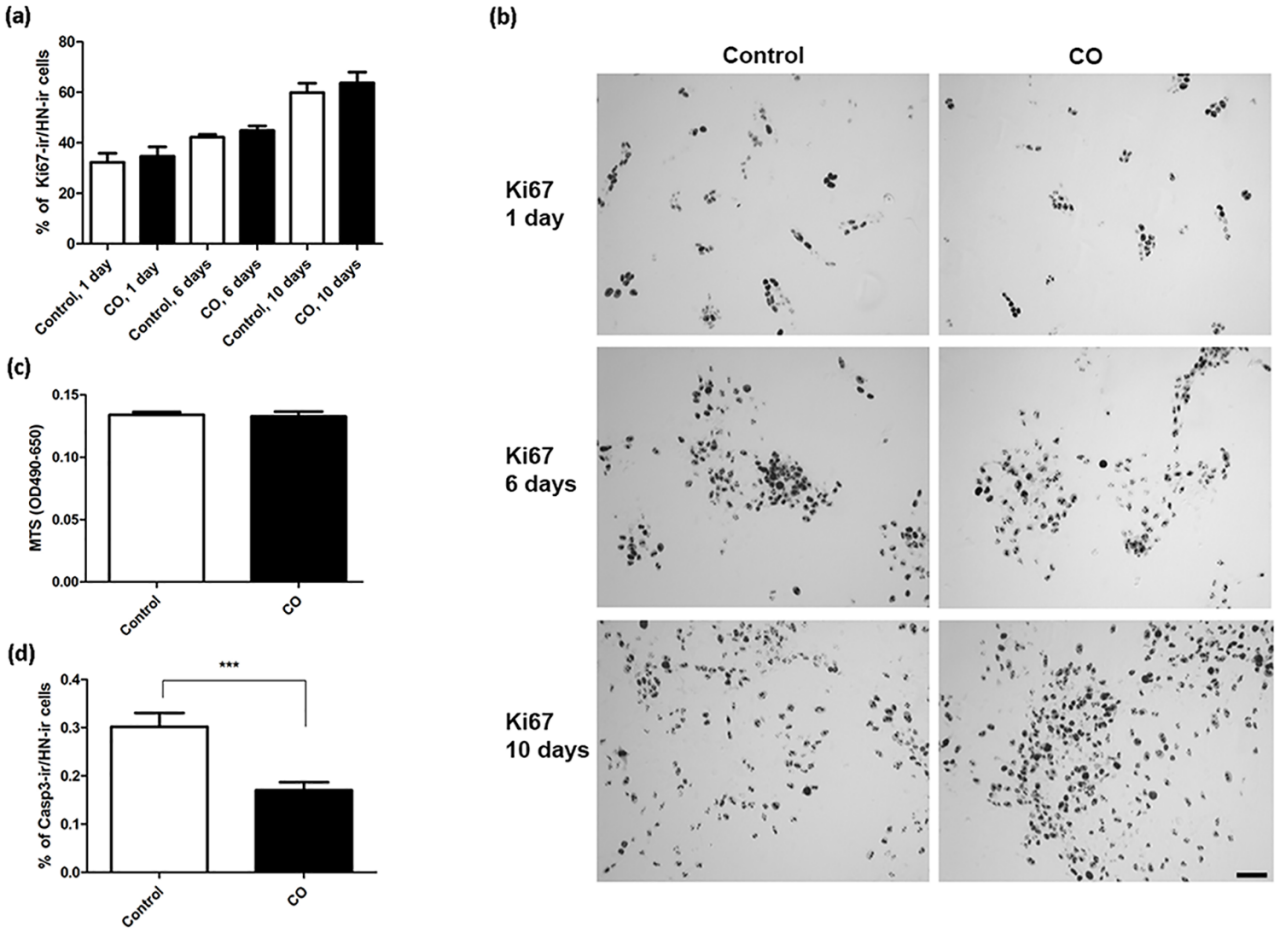
Effects of carbon monoxide (CO) treatment on proliferation and apoptosis. Assessment of proliferation in hVMbclX_L_ cells receiving 25 parts per million (ppm) CO at day 0 (30 min) and differentiated for 1, 6 and 10 days and apoptosis in cultures receiving 25 ppm CO treatment (30 min) at days 0 and 4 and differentiated for 6 days. Control cells followed the same protocol but received no CO treatment. (a) The percentage of Ki67-immunoreactive (Ki67-ir) cells of Human Nuclei (HN)-ir cells showed no difference between CO treatment and control cultures at any time-point (n = 12–14; two independent experiments). (b) Representative digital images of Ki67-ir cells receiving 25 ppm CO treatment at day 0 and differentiated for 1, 6 and 10 days. (c) Analyses of MTS reduction in cultures receiving 25 ppm CO treatment at days 0 and 4 and differentiated for 6 days did not differ between the groups. (d) The percentage of active/cleaved Caspase3 (Casp3)-ir apoptotic cells of total cells was significantly reduced for cultures receiving CO compared to controls (n = 26; two independent experiments). Data are expressed as mean±SEM (***p<0.001). (e) Digital images of Casp3-ir cells. Scale bar = 50 μm.

To address the effect of CO on cell proliferation, hVMbclX_L_ cells received a single dose of CO at day 0 (25 ppm; 30 min) and were differentiated for 1, 6 or 10 days. The relative content of proliferative cells did not differ between the groups at any time-point (control 1 day = 32.3±3.6; CO 1 day = 34.7±3.8; control 6 days = 42.3±1.1; CO 6 days = 44.9±1.8; control 10 days = 59.9±3.8; CO 10 days = 63.8±4.3; % Ki67-ir cells of total cells; mean±SEM; n = 12–14; two independent experiments). However, the overall percentage of Ki67-ir cells was found to increase during the differentiation from day 1 to 10 (Fig 5a). Representative photomicrographs of Ki67-ir cells are shown in Fig 5b. Further evaluation of metabolically active, viable cells in proliferation was performed by measuring MTS tetrazolium reduction in 6-day-old cultures receiving 25 ppm CO treatment (30 min) at days 0 and 4 compared to control. The analysis revealed no difference between the groups (Fig 5c). In summary, CO does not modulate cell proliferation during neuronal differentiation. To evaluate the potential effect of CO on apoptosis, 6-day-old cultures receiving 25 ppm CO treatment (30 min) at days 0 and 4 were immunostained for active/cleaved Casp3. The relative content of Casp3-ir cells was significantly reduced after CO treatment (control = 0.3±0.03; CO = 0.2±0.02 (p<0.001); % Casp3-ir cells of total cells; mean±SEM; n = 26; two independent experiments) (Fig 5d).

**Fig 6:**
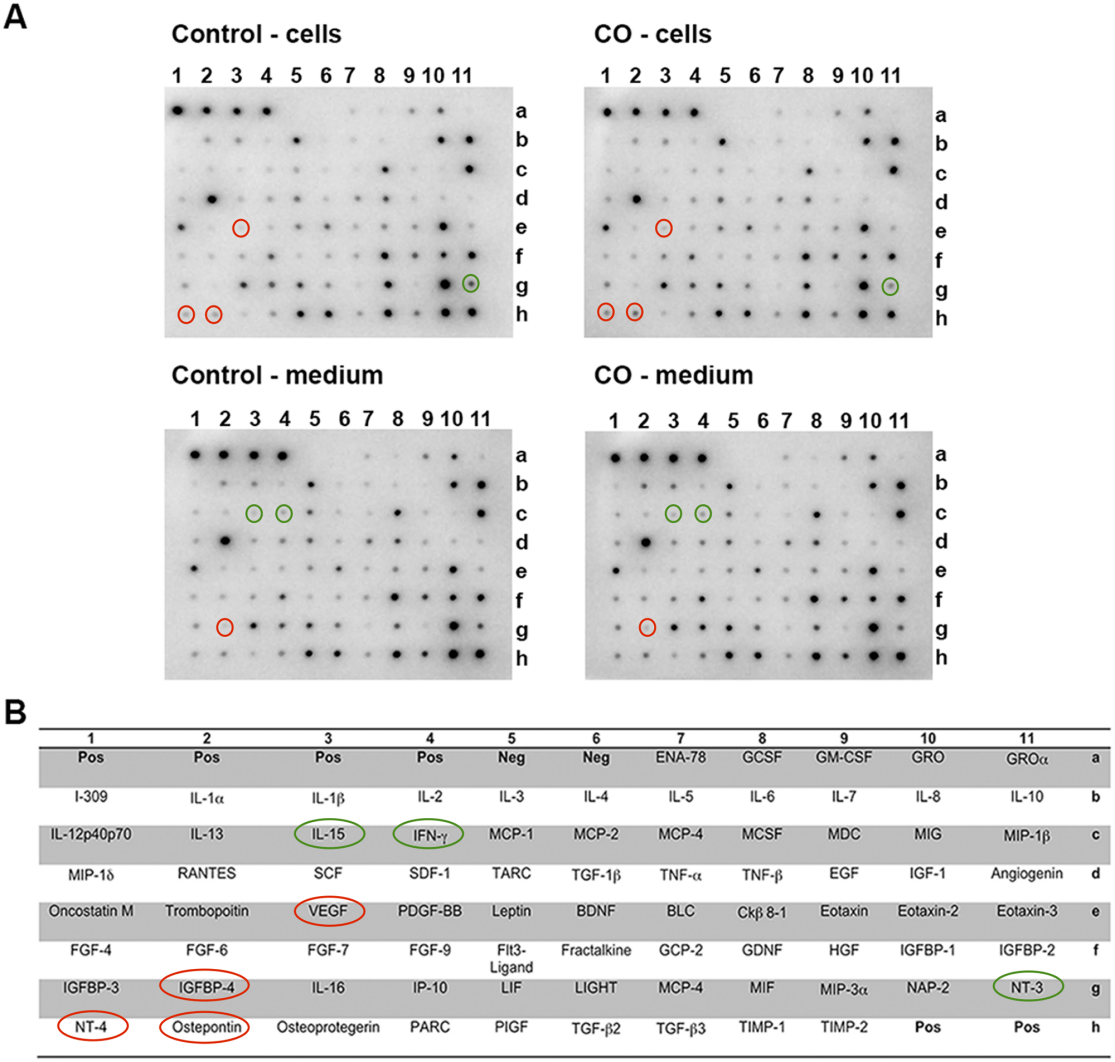
Effects of carbon monoxide (CO) treatment on cytokine profiles. Semi-quantitative expression profile of cytokines in cells (hVMbclX_L_) and conditioned medium from cultures receiving 25 parts per million (ppm) CO treatment (30 min) at days 0 and 4 and differentiated for 5 days compared to untreated controls. (a) Digital images of signal intensities for representative arrays containing 80 different cytokines plus positive and negative staining controls. Selected key changes in the arrays, representing the compiled dataset, are highlighted by red (up-regulation) and green circles (down-regulation). (b) Schematic overview illustrating the different cytokines. Densitometric analysis and comparison of signal intensities indicated an increase for vascular endothelial growth factor (VEGF), neurotrophin-4 (NT-4) and ostepontin and a relative reduction for neurotrophin-3 (NT-3) in cells receiving CO compared to controls. The content of cytokines in the medium indicated an increase for insulin-like growth factor binding protein 4 (IGFBP-4) and a reduction in interferon-γ (IFN-γ) and interleukin-15 (IL-15) for cultures treated with CO compared to control.

To address whether CO had an effect on cytokine profiles (conditioned culture medium/cell lysates), multi-cytokine analysis was performed using hVMbclX_L_ cells receiving 25 ppm CO (30 min) at days 0 and 4 and differentiated for 5 days versus untreated controls (Fig 6). Densitometric analysis indicated a reduction in the release of neurotrophin-3 (0.5 fold) and an increase in neurotrophin-4 (2.4 fold), vascular endothelial growth factor (2.3 fold), and osteopontin (1.9 fold) in cells receiving CO (Fig 6). The conditioned culture medium revealed a reduction in interleukin-15 (0.4 fold) and interferon-γ (0.5 fold) levels and an increase in insulin-like growth factor binding protein-4 (IGFBP-4; 1.7 fold) after CO treatment (Fig 6).

**Fig 7:**
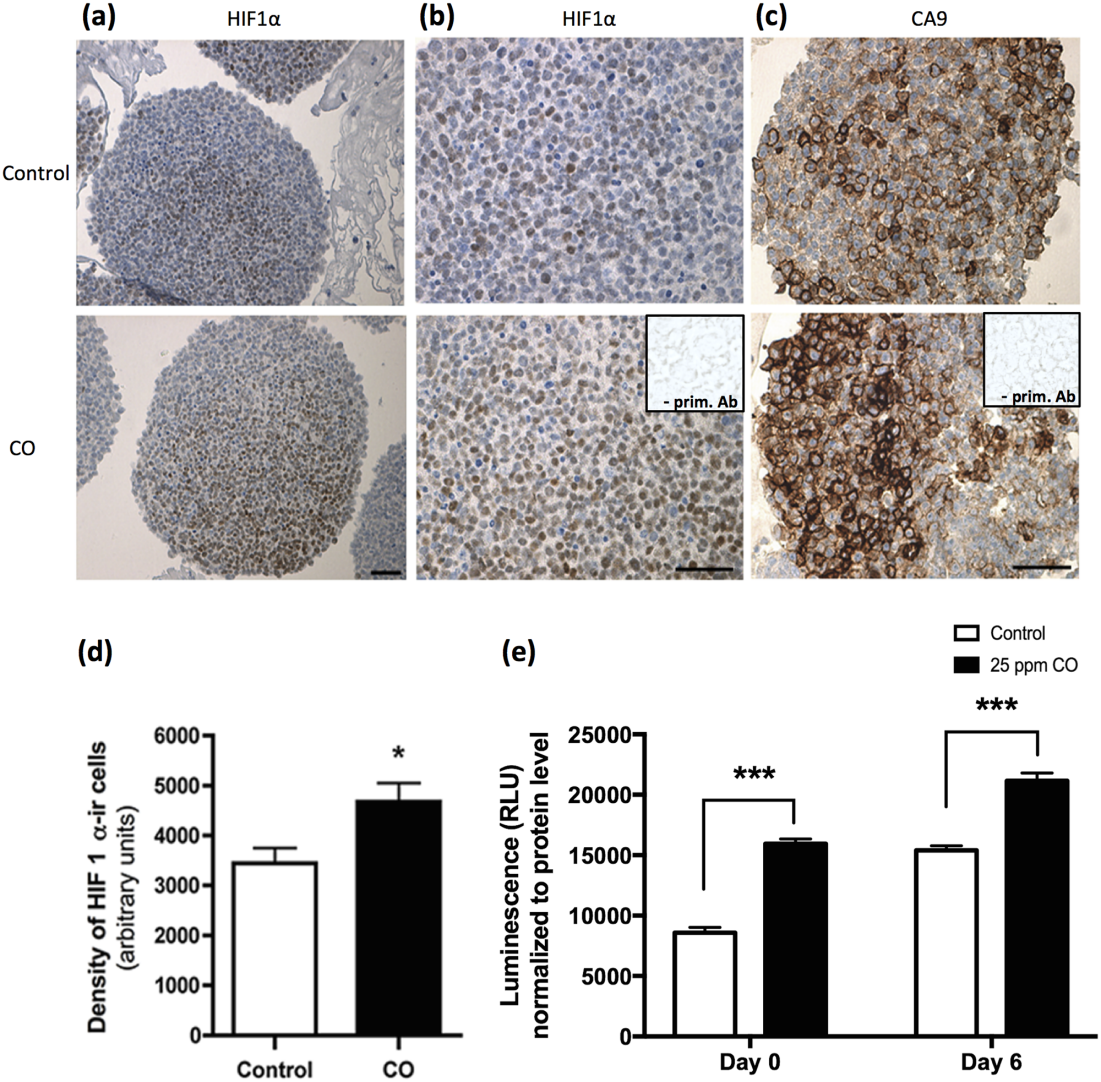
Effect of carbon monoxide (CO) treatment on stabilization of hypoxia inducible factor1α (HIF1α) and levels of reactive oxygen species (ROS). Assessment of HIF1α-immunoreactive (-ir) and carbonic anhydraseIX (CA9)-ir cells in hVMbclXL cultures propagated as neurospheres. Cells received 25 parts per million (ppm) CO (30 min) at days 0 and 4 and were grown for 6–9 days. (a, b) Representative photomicrographs of HIF1α-ir cells in thin sections from CO-treated and untreated neurospheres (insert: negative control; omission of primary antibody (Ab)). (c) Photomicrographs of CA9, a protein regulated by HIF1α, in sections from neurosphere cultures receiving CO versus control (insert: negative control). (d) Densitometric analysis of HIF1α-ir cells (values normalized to area of interest) revealed a significant increase in sections from cultures treated with CO compared to control. Scale bars = 50 μm. (e) Assessment of ROS in differentiating hVMbclXL monolayer cultures at day 0 (2 hrs after first CO exposure) and at day 6 (48 hrs after second CO treatment). At both time-points, ROS levels were significantly elevated (p<0.001) after CO treatment (25 ppm CO (30 min); RLU: relative light units).

To investigate whether CO treatment had an effect on HIF1α stabilization and the expression of a HIF1α regulated enzyme, CA9, hVMbclX_L_ cells were grown in non-adherent culture until neurospheres were visible. Neurospheres then received 25 ppm CO (30 min) at days 0 and 4 and were grown for 9 days (n = 16–18; two independent experiments). Immunostaining for HIF1α revealed a significant increase in the number of HIF1α-ir cells for cultures receiving CO compared to controls (Fig 7a and 7b–7d). Accordingly, neurospheres exposed to CO displayed more immunoreactivity to CA9 than untreated controls (Fig 7c).

To investigate whether the observed effect of CO also involved changes in levels of reactive oxygen species (ROS), ROS was assessed at 2 and 48 hrs after CO treatment (25 ppm, 30 min) (Fig 7e). The analysis showed that ROS levels were significantly elevated (p<0.001) at both time-points investigated (day 0, two hrs after CO exposure: control = 8593±437 relative light units (RLU) versus CO group = 15940±402 RLU and day 6, 48 hrs after CO exposure: control 15399±380 RLU versus CO group = 21165±643 RLU; mean±SEM; n = 6; two independent experiments).

In summary, the complex mechanisms underlying the observed effects of CO on stem cell differentiation involve a reduction of apoptotic cell death, a changed cytokine profile, stabilization of HIF1α and elevated ROS levels but do not influence cell proliferation.

## Discussion

In the present study, CO was generated by a decarbonylation reaction using the new CORM methyldiphenylsilacarboxylic acid (MePh_2_SiCO_2_H), along with the non-transition-metal activator potassium fluoride and dimethyl sulfoxide. This new strategy avoids the use of CO gas bottles, thus being more simple, safer and more cost-effective than previously described methods. To our knowledge, this is also the first paper to report a positive effect of CO on dopaminergic differentiation of human neural stem cells (NSCs). In brief, two stem cell lines were exposed to low levels of CO during their differentiation. Short-term CO treatment significantly increased both the numbers and relative yields of β-tubIII-ir neurons, suggesting that CO treatment stimulates neurogenesis. Moreover, the relative content of TH-ir neurons was significantly increased after CO exposure (Fig 3), which suggests that CO treatment favors induction or survival of the catecholaminergic phenotype. Exposure of hVMbclX_L_ cells to CO also increased the number of MAP2-ir neurons (Fig 4a), indicating that CO affects neuronal maturation. Control experiments using inactive CORMs (iCORMs) confirmed that the observed effects were due to the release of CO and not the reagents used to initiate the chemical reaction (S4 Fig).

Many biological effects of CO are associated with generation of low levels of reactive oxygen species, which act as signaling molecules [21–23,62–64]. CO-induced reactive oxygen species generation is mainly due to partial inhibition of cytochrome C oxidase [65–67]. Interestingly, stimulation of reactive oxygen species production is also important for cell signaling during neuronal differentiation and/or survival of embryonic stem cells, mesenchymal stem cells and neuronal progenitor cells [21,24,66,67].

In our study, CO treatment did not alter the total number of cells. Furthermore, the MTS analysis used to assess cell proliferation and viability revealed no change after CO treatment, which was in line with the unchanged content of HN-ir cells (Fig 5). Thus, it is unlikely that the applied CO concentrations influenced cell proliferation or were toxic to the cells. This is to some extent in accordance with studies showing an anti-proliferative effect of CO in other tissues, including vascular smooth muscle cells and T-lymphocytes, through activation of mitogen-activated protein kinases and the cell cycle inhibitor p21 [16,68,69].

Interestingly, studies exposing NSCs to low oxygen tension have reported both increased cell proliferation and dopaminergic differentiation [14,70]. In the present study using CO exposure in ambient oxygen, dopaminergic differentiation was increased without an increase in cell proliferation, which suggests other underlying mechanisms than those triggered by low oxygen.

Previous studies have shown an anti-apoptotic effect of CO treatment on fibroblasts, endothelial cells, astrocytes and cerebellar granule cells [21,23,71,72]. To address whether a similar effect was present in hVMbclX_L_ cells, cultures were immunostained for active/cleaved Casp3. The relative content of Casp3-ir cells was significantly reduced after CO, indicating that CO has an anti-apoptotic effect. However, the number of Casp3-ir cells was very low most likely due to the over-expression of the anti-apoptotic protein BclX_L_, which should be taken into account. Interestingly, Almeida and colleagues have recently demonstrated that CO increases neuronal differentiation in hippocampal slice cultures, NT2 and SH-Y5Y cells by limiting apoptosis [35]. The specific molecular mechanism by which CO suppresses apoptosis was beyond the scope of the present study, but it is likely to involve p38 MAP kinases.

For characterization of the catecholaminergic cell population obtained after CO treatment, the expression of catecholaminergic/midbrain-specific genes (TH, Nurr1, DAT and DBH) was assessed by real-time Q-PCR (Fig 4c). Nurr1 is involved in maintenance of midbrain dopaminergic activity and is related to dopaminergic differentiation since Nurr1-null mouse-derived NSCs fail to differentiate and express TH [73,74]. In the present study, Nurr1 expression was increased after CO exposure, which is in line with the observed increase in TH expression and release of dopamine and homovanilic acid. This could indicate that a substantial fraction of neurons was dopaminergic.

The levels of dopamine in neuronal cells can be modulated by the activity of DAT and DBH. DAT is responsible for dopamine transport from the synaptic cleft, and surprisingly it was down-regulated after CO treatment. This may be due to the significant increase in the pool of free dopamine after CO treatment and/or due to the artificial *in vitro* conditions. The observed increase in DBH after CO treatment, which catalyses the conversion of dopamine into noradrenaline, may simply reflect the rise of intracellular dopamine levels as a result of TH up-regulation.

Semi-quantitative cytokine profiling of cell lysates and conditioned culture medium showed an increase in vascular endothelial growth factor for CO-treated cultures (cell lysates) compared to controls (Fig 6). This is consistent with other studies demonstrating that CO elevates vascular endothelial growth factor levels in astrocytes and cardiomyocytes [75–77]. Interestingly, a reduction in neurotrophin-3 and an increase in neurotrophin-4 levels were found in cell lysates from cultures receiving CO treatment. No studies have investigated the effect of CO treatment on neurotrophin-3 and neurotrophin-4, but both neurotrophins have been reported to be involved in neuronal growth, synapse formation, maturation and plasticity. Moreover neurotrophin-3 is expressed in NSCs, stimulating their neuronal differentiation and survival [78–80]. The down-regulation of neurotrophin-3 may reflect the observed stimulatory effect of CO on neurogenesis and cell maturation leading to a reduction in the pool of NSCs. On the other hand the observed up-regulation of neurotrophin-4 could potentially stimulate further maturation and growth of cells, which, at day 5, are still undergoing differentiation. An increase in osteopontin expression was also found in CO-treated cultures, which to some extent is consistent with another study, showing that heme oxygenase-1 activity increased osteopontin expression and promoted differentiation of odontoblasts [81].

The cytokine profiling of conditioned culture medium revealed changes for interleukin-15, interferon-γ and IGFBP-4 after CO treatment (Fig 6). The decrease in interleukin-15 observed for CO-treated cultures could indicate that CO exhibit an anti-inflammatory effect on NSCs. Previous studies have demonstrated that interleukin-15 is a pro-inflammatory cytokine present in both NSCs and differentiated neurons during inflammation, but it has also been reported that decreased levels of interleukin-15 *in vivo* leads to an increase in cell differentiation and reduction in cell proliferation [82–84]. Interestingly, a study culturing rat NSCs showed that interleukin-15 treatment reduced the number of MAP2-ir neurons thus inhibiting neuronal maturation [85]. The observed decrease of interleukin-15 found in our study may therefore have contributed to increased cell maturation as shown by the increased number of MAP2-ir neurons found after treatment. The reduction in interferon-γ in cultures receiving CO also suggests an anti-inflammatory role of CO since existing literature describe pro-inflammatory characteristics of interferon-γ [32].

Previous studies have shown that IGFBP-4 plays a role in the developing brain by stimulating neuronal differentiation of NSCs. In the present study the increase in IGFBP-4 in cultures receiving CO could indicate that CO signals through IGFBP-4 to promote cell differentiation [86,87].

Culturing of human NSCs at low oxygen tension has been shown to favor their dopaminergic differentiation [59,70,88,89]. During fetal development and even in the adult brain the physiological oxygen tension is relatively low (1–5%) [90]. Low oxygen tension stabilizes the transcription factor HIF1α, which leads to up-regulation vascular endothelial growth factor and erythropoietin [88,91–94]. Previous studies have suggested that also CO can stabilize HIF1α [52,75,95]. In the present study, immunostaining for HIF1α revealed an increase in HIF1α expression in cultures receiving CO compared to controls. This, together with the detected increase in vascular endothelial growth factor, could indicate that the observed effect CO on NSCs to some extent mimics effects of low oxygen, by stabilizing HIF1α [94,96,97] (Fig 7). However, our additional finding of a significant and long-lasting elevation in ROS levels after treatment clearly indicates that CO has a more complex mechanism of action.

## Conclusion

Short-term treatment of differentiating human neural stem cells with a low dose of CO, produced by CO-releasing compounds, represents an efficient, simple, cost-effective and safe method for *in vitro* derivation of viable dopaminergic neurons with midbrain characteristics. This finding may have implications for the derivation of cells for experimental studies and future development of donor cells for potential transplantation in Parkinson’s disease.

## Supporting information

S1 Fig Repeated measure of carbon monoxide (CO) levels in the CO chamber during the 30 min exposure period. Measurements visualized in the figure represent data from the analysis of 4 different CO concentrations (12.5–100 parts per million (ppm)). Data are expressed as mean±SEM (12.5 ppm: n = 4; 25 ppm: n = 5; 50 ppm: n = 5; 100 ppm: n = 5 at each time point; 4–5 independent experiments).
(TIF)

S2 Fig Analysis of cells by a NucleoCounter^®^ NC-200. Neural stem cells (hVMbclX_L_) were dissociated using trypsin/EDTA, centrifuged for 5 min at 800 rpm and 4°C, resuspended in culture medium and loaded on the automatic cell analyzer. (a) Data on cell viability, cell diameter and density. (b) Image of cells counted in the sample. (c) Graph representing cells stained with Acridine Orange (AO), marking all viable and non-viable cells and their distribution in a Via1-Cassette, revealed that 90% of the cells were located in the squared area of counting. (d,e) The intensity and location of cells stained with AO. (f) Non-viable cells stained with 4’,6-diamidino-2-phenylindole and their distribution in the Via1-Cassette. (g,h) The intensity and location of cells stained with 4’,6-diamidino-2-phenylindole.
(TIF)

S3 Fig Effects of carbon monoxide (CO) treatment on neuronal differentiation of neural stem cells. Human REN VM cells were plated in laminin-coated trays at a density of 26,000 cells/cm^2^ and differentiated for 6 days. One group of cultures was treated with 25 parts per million (ppm) CO for 30 min at days 0 and 4. Control cells received no CO treatment. (a) Quantification of β-tubulinIII-immunoreactive (β-tubIII-ir) neurons showed a significant increase for CO-treated cultures compared to control. (b) The percentage of β-tubIII-ir neurons of human nuclei (HN)-ir cells (total cells) was significantly higher for the CO treatment group compared to control (n = 10). Data are expressed as mean±SEM (***p<0.001). (c,d) Representative digital photomicrographs of β-tubIII-ir neurons and HN-ir cells in CO-treated and control cultures. Scale bar = 50μm.
(TIF)

S4 Fig Test of inactive carbon monoxide releasing molecules (iCORMs) on dopaminergic differentiation. To validate that the observed effect of the CORMs on dopaminergic differentiation was mediated by CO, hVMbcl-xl cells were exposed to iCORMs (potassium flouride, 1,25 mg; dimethyl sulfoxide, 0.25 ml) for 30 min at days 0 and 4 and differentiated for 6 days. Cultures kept under the same conditions but without exposure to CORMs served as a reference and additional control. At day 6, cultures were immunostained for tyrosine hydroxylase (TH) and human nuclei (HN; total cells). (a) The relative content of TH-immunoreactive (-ir) neurons, revealed no significant difference between the iCORM exposure group and the untreated control group (n = 11–20). Data are expressed as mean±SEM.
(TIF)
