# Supplementary figures and images for: Intermittent, low dose carbon monoxide exposure enhances survival and dopaminergic differentiation of human neural stem cells
Source: PLoS One. 2018 Jan 16;13(1):e0191207. doi: 10.1371/journal.pone.0191207 (PMC5770048; doi:10.1371/journal.pone.0191207)

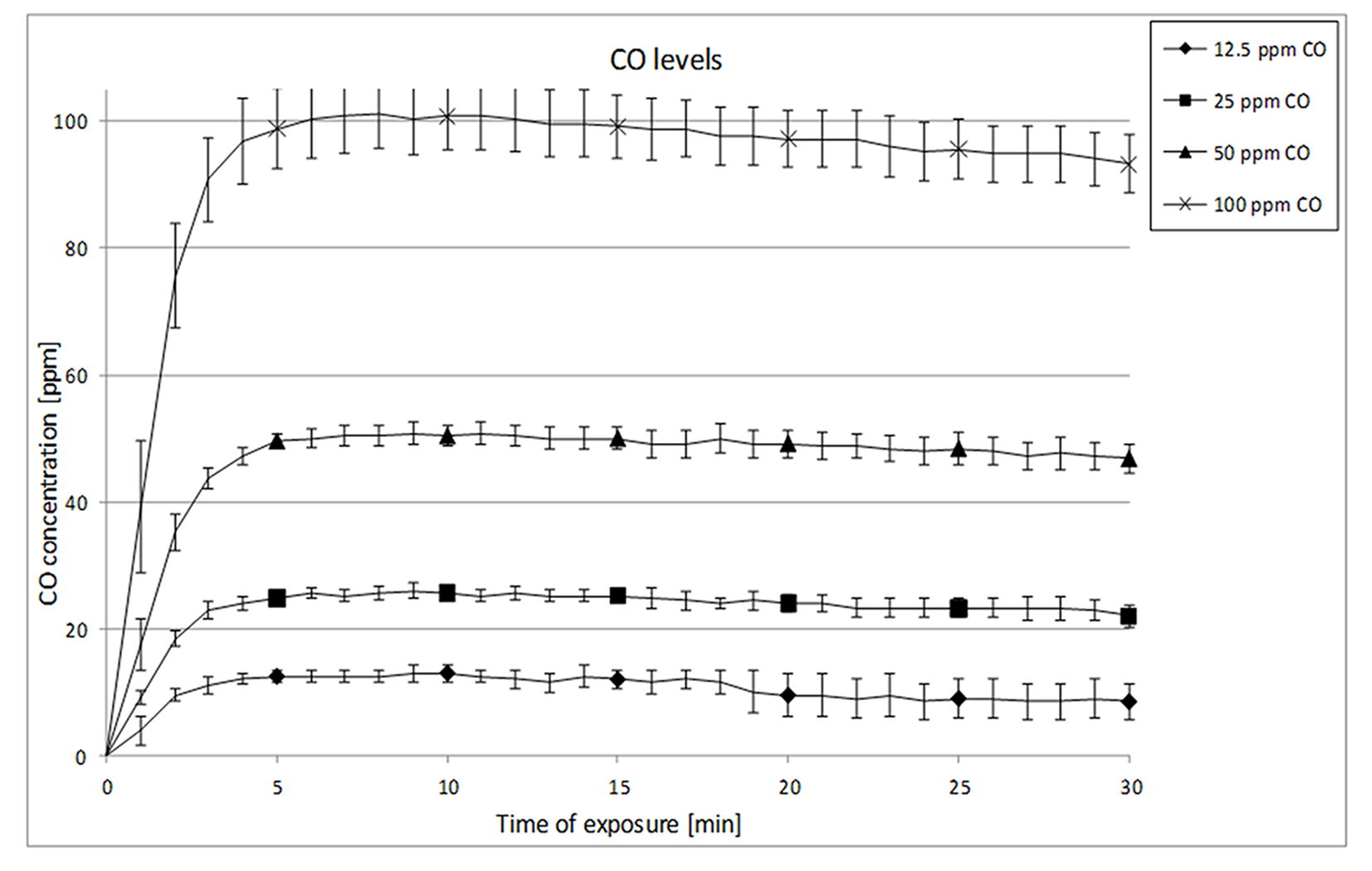

Supplement: S1 Fig — Measurements visualized in the figure represent data from the analysis of 4 different CO concentrations (12.5–100 parts per million (ppm)). Data are expressed as mean±SEM (12.5 ppm: n = 4; 25 ppm: n = 5; 50 ppm: n = 5; 100 ppm: n = 5 at each time point; 4–5 independent experiments). (TIF) [file pone.0191207.s001.tif]

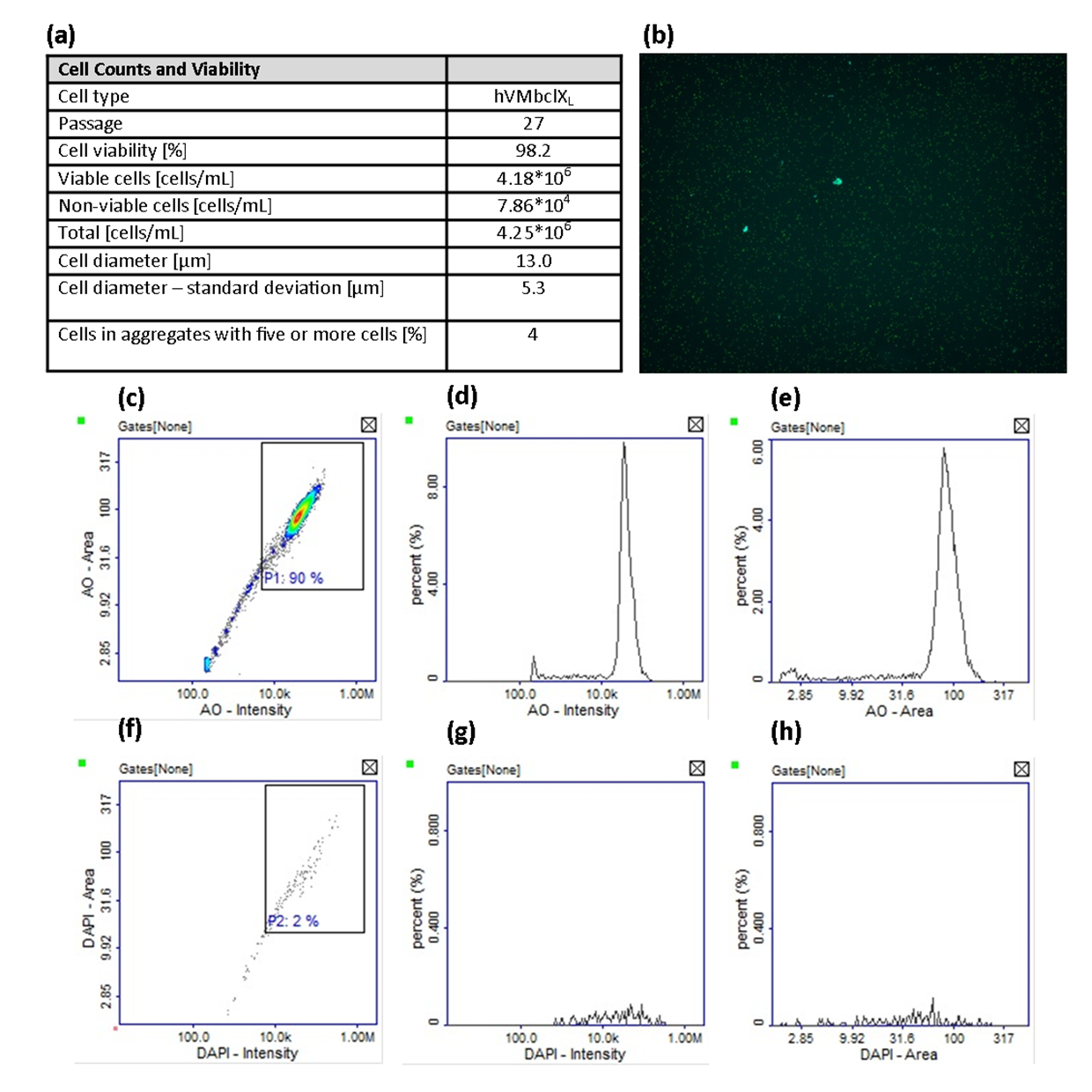

Supplement: S2 Fig — Neural stem cells (hVMbclXL) were dissociated using trypsin/EDTA, centrifuged for 5 min at 800 rpm and 4°C, resuspended in culture medium and loaded on the automatic cell analyzer. (a) Data on cell viability, cell diameter and density. (b) Image of cells counted in the sample. (c) Graph representing cells stained with Acridine Orange (AO), marking all viable and non-viable cells and their distribution in a Via1-Cassette, revealed that 90% of the cells were located in the squared area of counting. (d,e) The intensity and location of cells stained with AO. (f) Non-viable cells stained with 4’,6-diamidino-2-phenylindole and their distribution in the Via1-Cassette. (g,h) The intensity and location of cells stained with 4’,6-diamidino-2-phenylindole. (TIF) [file pone.0191207.s002.tif]

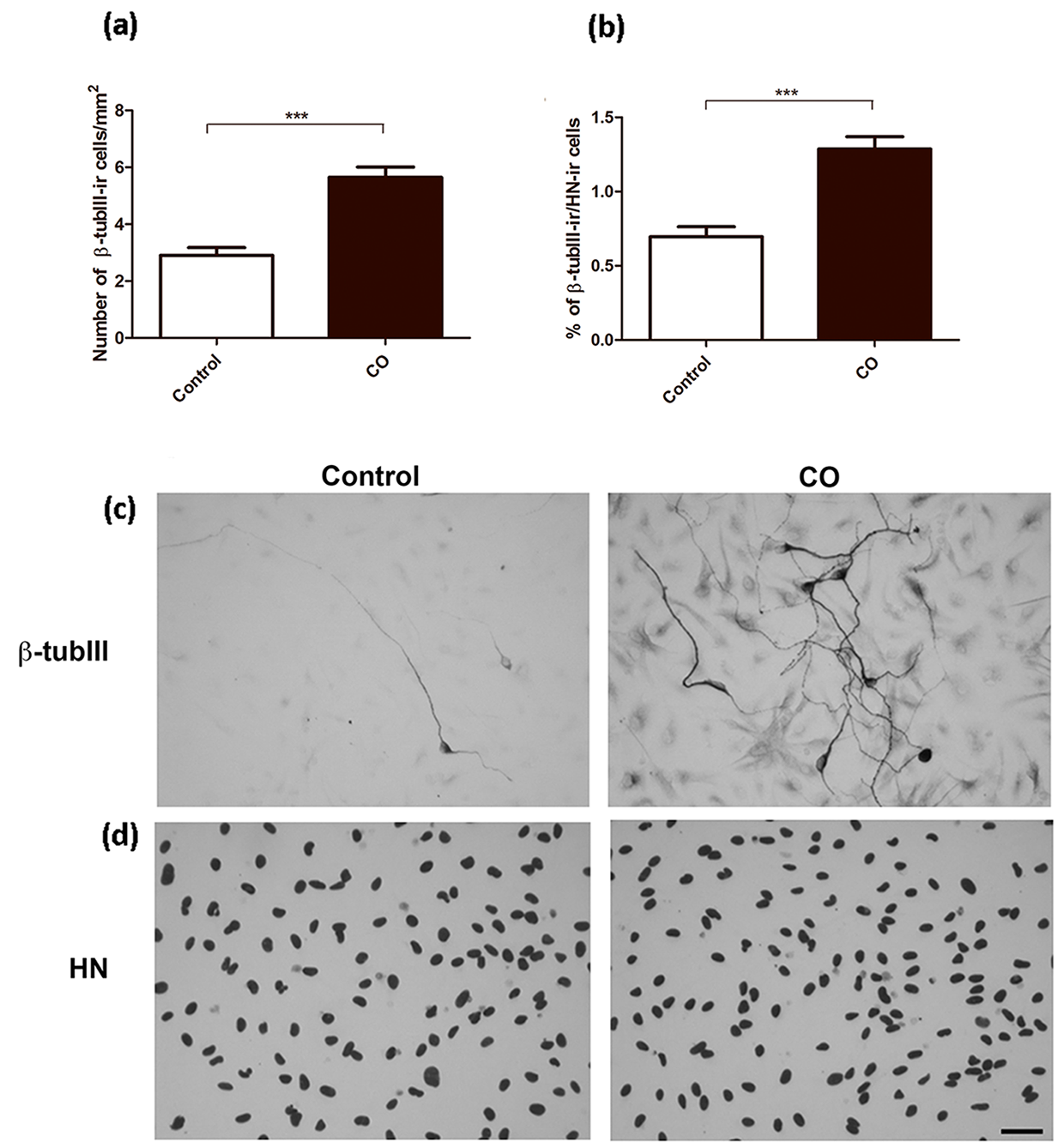

Supplement: S3 Fig — Human REN VM cells were plated in laminin-coated trays at a density of 26,000 cells/cm2 and differentiated for 6 days. One group of cultures was treated with 25 parts per million (ppm) CO for 30 min at days 0 and 4. Control cells received no CO treatment. (a) Quantification of β-tubulinIII-immunoreactive (β-tubIII-ir) neurons showed a significant increase for CO-treated cultures compared to control. (b) The percentage of β-tubIII-ir neurons of human nuclei (HN)-ir cells (total cells) was significantly higher for the CO treatment group compared to control (n = 10). Data are expressed as mean±SEM (***p<0.001). (c,d) Representative digital photomicrographs of β-tubIII-ir neurons and HN-ir cells in CO-treated and control cultures. Scale bar = 50μm. (TIF) [file pone.0191207.s003.tif]

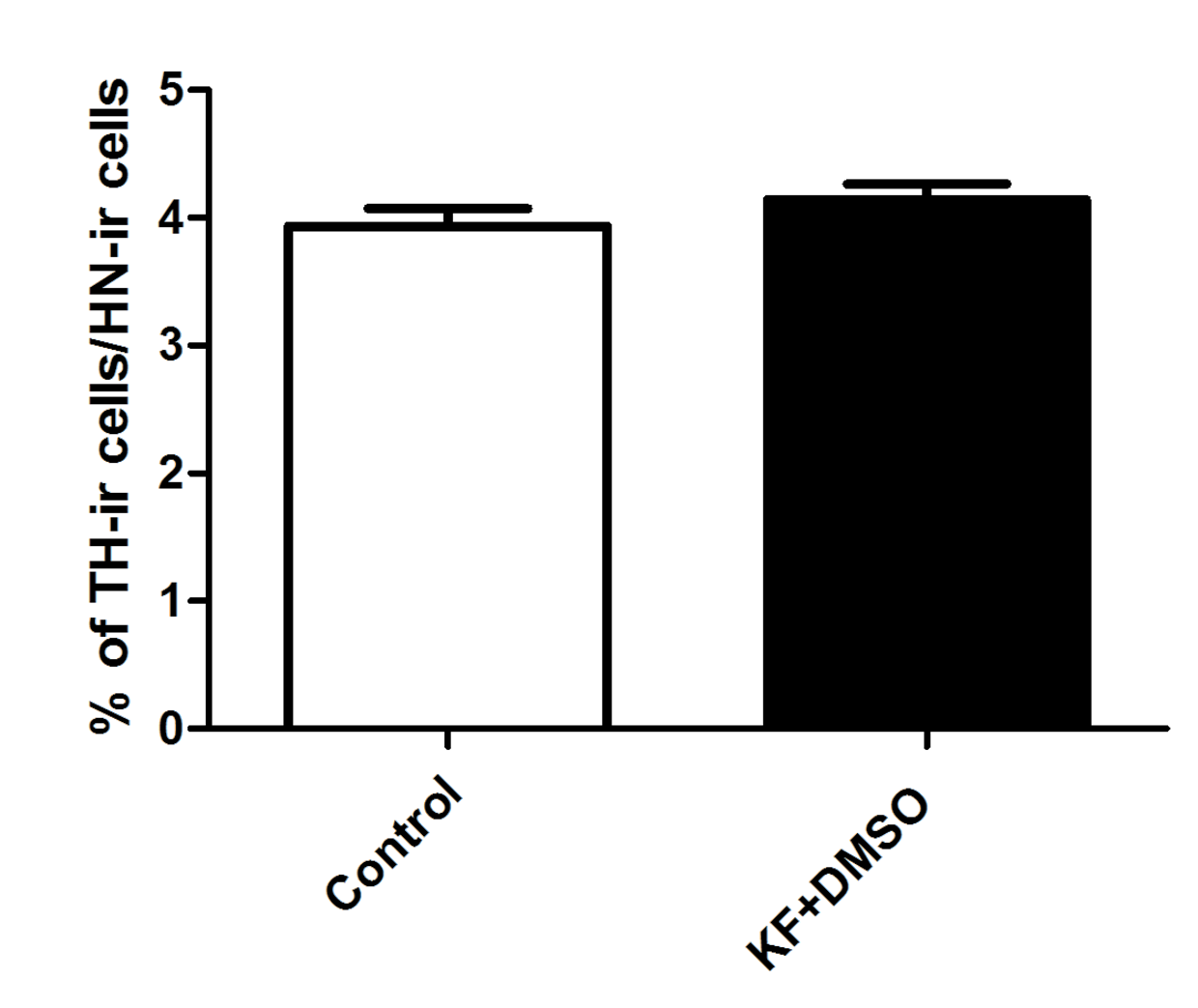

Supplement: S4 Fig — To validate that the observed effect of the CORMs on dopaminergic differentiation was mediated by CO, hVMbcl-xl cells were exposed to iCORMs (potassium flouride, 1,25 mg; dimethyl sulfoxide, 0.25 ml) for 30 min at days 0 and 4 and differentiated for 6 days. Cultures kept under the same conditions but without exposure to CORMs served as a reference and additional control. At day 6, cultures were immunostained for tyrosine hydroxylase (TH) and human nuclei (HN; total cells). (a) The relative content of TH-immunoreactive (-ir) neurons, revealed no significant difference between the iCORM exposure group and the untreated control group (n = 11–20). Data are expressed as mean±SEM. (TIF) [file pone.0191207.s004.tif]
